# Obstructive Sleep Apnea Syndrome Comorbidity Phenotypes in Primary Health Care Patients in Northern Greece

**DOI:** 10.3390/healthcare10020338

**Published:** 2022-02-10

**Authors:** Panagiota K. Ntenta, Georgios D. Vavougios, Sotirios G. Zarogiannis, Konstantinos I. Gourgoulianis

**Affiliations:** 1 Department of Respiratory Medicine, Faculty of Medicine, School of Health Sciences, University of Thessaly, 41500 Larissa, Greece; yiotantenta@gmail.com (P.K.N.); gvavougyios@uth.gr (G.D.V.); kgourg@uth.gr (K.I.G.); 2 Department of Physiology, Faculty of Medicine, School of Health Sciences, University of Thessaly, 41500 Larissa, Greece

**Keywords:** comorbidities, Greece, OSAS, phenotyping, primary health care

## Abstract

Background: Obstructive sleep apnea syndrome (OSAS) is a significant public health issue. In the general population, the prevalence varies from 10% to 50%. We aimed to phenotype comorbidities in OSAS patients referred to the primary health care (PHC) system. Methods: We enrolled 1496 patients referred to the PHC system for any respiratory- or sleep-related issue from November 2015 to September 2017. Some patients underwent polysomnography (PSG) evaluation in order to establish OSAS diagnosis. The final study population comprised 136 patients, and the Charlson comorbidity index was assessed. Categorical principal component analysis and TwoStep clustering was used to identify distinct clusters in the study population. Results: The analysis revealed three clusters: the first with moderate OSAS, obesity and a high ESS score without significant comorbidities; the second with severe OSAS, severe obesity with comorbidities and the highest ESS score; and the third with severe OSAS and obesity without comorbidities but with a high ESS score. The clusters differed in age (*p* < 0.005), apnea–hypopnea index, oxygen desaturation index, arousal index and respiratory and desaturation arousal index (*p* < 0.001). Conclusions: Predictive comorbidity models may aid the early diagnosis of patients at risk in the context of PHC and pave the way for personalized treatment.

## 1. Introduction

Obstructive sleep apnea syndrome (OSAS) is a disease characterized by recurrent episodes of partial or complete collapse of the upper airway. It occurs during sleep despite the documented ongoing effort to breath. This leads to a partial reduction in airflow, followed by arousal, with an abrupt reduction in blood oxygen saturation (hypopneas). A complete cessation of airflow lasting at least 10 s during sleep is defined as apnea. This pattern can occur many times during the night [1].

OSAS prevalence is estimated to be approximately 5–10% in the general population, regardless of race and ethnicity, with certain subgroups of the population bearing higher risk [2]. An estimated 10% of middle-aged men and 3% of middle-aged women suffer from moderate/severe disease [3].

Early OSAS diagnosis can make a significant contribution to reducing comorbidity, healthcare costs, mortality rates and facilitating the reference for medical services. Still, few studies have investigated the early detection of OSAS in patients attending primary health care (PHC) services either due to snoring and/or sleep interruption and daytime sleepiness. Predictive comorbidity models enrich our knowledge about OSAS heterogeneity and aid in the early diagnosis of patients at risk, facilitating better individualized treatment approaches for patients. Some years ago, we were the first to employ, categorical principal component analysis (CATPCA) in combination with cluster analysis in an OSAS population in order to detect comorbidity phenotypes [4]. Our scope was to identify the interplay between OSAS and its comorbidities, an approach that had served well in phenotyping in other areas and that also gained popularity after our study in the OSAS field [5,6,7,8,9,10,11].

The aim of the current study was to evaluate our previously published CATPCA and TwoStep cluster (TSC) predictive comorbidity phenotyping model in OSAS patients who underwent polysomnography (PSG) after referral from the Greek PHC services.

## 2. Materials and Methods

### 2.1. Study Population

We performed an epidemiological observational study to investigate the relationship between OSAS, concomitant diseases and the characteristics of the studied PHC population. We screened 1496 patients referred to the PHC system in the northern part of Greece for any respiratory or sleep problem from November 2015 to September 2017. The study population consisted of adults, 18 to 65 years of age, who had previously undergone a full standard PSG in the Sleep Unit of the “AGIOS PAVLOS” General Hospital. Patients previously diagnosed with respiratory failure were excluded from our study. We designed a database that included patient demographics and anthropometric and socio-economic data, along with behavioral characteristics, clinical history, and Epworth Sleepiness Scale and Berlin Questionnaire results, and all parameters were included in the patients’ overnight PSGs in the laboratory. From the initially screened population, 136 patients were included in the final analysis. The study was conducted according to the guidelines of the Declaration of Helsinki and approved by the Institutional Review Board of the 4th HEALTH REGION–MACEDONIA & THRACE (21990/5 July 2016).

### 2.2. OSAS Definition Severity and Diagnosis

A common measurement of sleep apnea is the apnea–hypopnea index (AHI), which is the number of apneas and hypopneas that occur per hour of sleep, and it is used to grade the degree of OSAS severity [12]. In our study, the determination of AHI was achieved after a full-night PSG in the laboratory. According to the American Academy of Sleep Medicine (AASM), there are four types of OSAS [13,14]:

1. Normal (AHI < 5);
2. Mild (AHI ≥ 5–14);
3. Moderate (AHI ≥ 15–29);
4. Severe OSAS (AHI ≥ 30).

### 2.3. Polysomnography (PSG)

PSG is considered the “gold-standard” for sleep disorder diagnosis and requires an overnight stay in a sleep laboratory. Laboratory-based full-night PSG was performed in the studied population and included the monitoring of sleep state through the use of electroencephalography (EEG), electrooculography (EOG), electromyography (EMG), electrocardiography (ECG), oral thermistor, sound probe around the neck for snoring measurement, pulse oximetry-determined changes in blood oxygen levels and body position, and the respiratory effort was measured with the use of abdominal and thoracic belts. Arousals were measured as sudden shifts in brain wave activity. Apnea was defined as a pause in respiration nasal airflow using pressure transducers for at least 10 s. Apneas were further classified as obstructive, central or mixed based on whether the effort to breath was present during the event. Hypopnea was defined as a reduction in ventilation of at least 30% from the baseline in airflow reduction for >10 s. This results in a decreased arterial saturation associated with at least 4% oxygen desaturation due to partial airway obstruction [15].

### 2.4. Questionnaires

In addition to PSG, screening tools, such as questionnaires, indicate the risk of patients for OSAS. They are simple, cost effective and validated tools recommended for the initial screening of OSAS [15]. The Epworth Sleepiness Scale (ESS) consists of eight different situations, and the questionnaire asks the subject to rate the probability of falling asleep in specific situations. Patients with scores ≥ 11 and experiencing involuntary sleepiness during activities that require more active attention, such as talking or driving, are suggested to have excessive daytime sleepiness [14,16]. The Berlin Questionnaire (BQ) has three parts. The survey items address the presence and frequency of snoring behavior, daytime sleepiness or fatigue and history of obesity or hypertension [15].

### 2.5. Comorbidity Assessment

Comorbidity assessment is an important component of health services research and an inevitable clinical prognostic factor. Comorbidity may impact treatment, prognosis and quality of care assessment. In our study, the severity of comorbidity was determined based on the adjusted Charlson comorbidity index (CCI) score. The CCI score was calculated for the studied population based on an algorithm formatted as a Microsoft Excel Macro, which provides a rapid method for calculating the CCI score. The CCI score was developed by Hall et al. in 1987 and predicts the one-year mortality for a patient who may have a range of comorbid conditions (index consists of 19 medical conditions). Each condition is assigned a score of 1, 2, 3, or 6, with total scores ranging from 0 to 37. Comorbidity, in our study, was defined in terms of the absence or presence of one or more conditions included in and scored by the CCI criteria [17].

### 2.6. Statistical Analysis

The statistical analysis was performed using the IBM SPSS Statistics 21.0 software package. Data normality was assessed by the Kolmogorov–Smirnov test. Data are presented as mean ± standard deviation (SD) for data with normal distribution, and as median with interquartile ranges in parenthesis for skewed data. Independent samples *t* test or Mann–Whitney U test was used where appropriate. Categorical variables are expressed as percentages, and the chi-square or Fischer’s exact test was used. Differences between clusters were tested by one-way ANOVA analysis with post-hoc Bonferroni correction. *p* < 0.05 is considered significant.

### 2.7. Categorical Principal Component Analysis (CATPCA)

CATPCA is a variant of the principal component analysis (PCA). This method is the nonlinear equivalent of the standard PCA and reduces the observed set of variables into a smaller set of uncorrelated variables called principal components, which represent most of the information found in the original variables. The most important advantages of nonlinear over linear PCA are that it incorporates multivariate variables and that it can handle and discover nonlinear relationships between variables [18]. The method is most useful when many variables prohibit an effective interpretation of the relationships between objects. It reduces the dimensionality of a set of variables while accounting for as much of the variation as possible, and this optimal scaling allows better performance. By reducing the dimensionality, the clustering process is facilitated. Categorical variables are optimally quantified in the specified dimensionality. As a result, nonlinear relationships between variables can be modeled. CATPCA maximizes the correlations of the object scores with each of the quantified variables for the number of components (dimensions) specified to be used as clustering variables. This method was used on the components of the CCI.

### 2.8. TwoStep Clustering

Subsequently, components extracted from the CATPCA were used as clustering variables along with the AHI. For this, the SPSS TwoStep cluster (TSC) method was used. TSC is a scalable cluster analysis algorithm designed to handle very large mixed datasets and to reveal data groups (clusters) within a dataset that would not otherwise be apparent. TSC integrates a hierarchical and partitioning clustering algorithm, adding attributes to cluster objects. This method defines the relationships among items and improves the weaknesses of applying a single clustering algorithm. Clusters are categories of items with many features in common.

The TSC method is divided into the following two steps: Step 1: the pre-cluster step identifies regions with dense intercorrelations in the input variables and produces primary sub-clusters [19]. Step 2: these initial sub-clusters resulting from the first step are merged into the final clusters using the hierarchical agglomerative clustering method. Determining the optimal number of clusters in a data set is a fundamental issue in partitioning clustering; the TwoStep algorithm automatically incorporates the Bayesian information criterion (BIC) and the change in the distance measurement by also calculating the log likelihood ratio distance [20].

Finally, a post-hoc test provided by the TSC method is the average silhouette coefficient, which is a measure that indicates how similar an object is to its own cluster (cohesion) compared to other clusters (separation). The silhouette ranges from −1 (indicating a very poor model) to +1 (indicating that the object is well matched to its own cluster and poorly matched to neighboring clusters). An average silhouette greater than 0.5 indicates a reasonable partitioning of data [21]. In our study, the optimal cutoff was set at >0.85 when comparing the differentiation between clusters.

## 3. Results

### 3.1. Participant Characteristics

Among the 288 participants, 115 patients were diagnosed with other sleeping breathing disorders, 28 declined to undergo PSG, and 9 recordings were technically unacceptable. Thus, a total of 136 patients were included in our study. In Table 1, the demographics and the respiratory and sleep apnea characteristics of the study participants are shown.

### 3.2. Cluster Analysis

CATPCA produced three components (Table 2 represents Cronbach’s alpha value) that were used with AHI staging as clustering variables in the TSC technique. Applying this method, three distinct clusters were identified from the analysis.

Clusters can correspond to phenotypes. The first phenotype corresponds to moderate OSAS, obesity and a high ESS score without significant comorbidities; the second to severe OSAS, severe obesity with comorbidities and the highest ESS score; and the third to severe OSAS and obesity without comorbidities but with a high ESS score. The clusters differed significantly in age (*p* < 0.005), apnea–hypopnea index (AHI), oxygen desaturation index (ODI), arousal index (AI) and respiratory and desaturation arousal index (*p* < 0.001) (Table 3, Table 4 and Table 5).

Total Cronbach’s alpha and total variance explained based on cumulative eigenvalue were determined for each scaled model in comparison to the previous model. The objective of this evaluation was to maintain internal consistency among comorbidity features, while aiming prospectively for an increase in variance explained by the scaled model.

## 4. Discussion

Although OSAS public awareness and its associated health consequences have increased, the prevalence of OSAS continues to rise, and a significant part of the affected population remains undiagnosed. Several studies have shown that a phenotyping approach is important in early detection and provides more adapted treatment to the patient’s needs. The identification of subgroups based on comorbidities and symptoms is crucial to understand OSAS causality and to develop management strategies customized for each subgroup. Such approaches have not been validated in the context of a community-based population [22]. Our study aimed to apply an OSAS comorbidity phenotyping approach previously developed by our group in a sample of the primary health care population [4].

This study placed emphasis on the standardized definition of comorbidity and OSAS severity based on AHI. TSC revealed three distinct clusters referred to as moderate-to-severe OSAS in our study population. Comorbidity scores were segregated into the severe OSAS cluster without comorbidities and severe OSAS with comorbidities. Our results are in line with those of the study by Vavougios et al. [4], although we found three distinct clusters out of six, which was maybe due to the lower number of patients. Specifically, Vavougios et al. found six distinct clusters, where one corresponded to healthy subjects, one corresponded to mild OSAS without comorbidities, two corresponded to moderate OSAS with and without comorbidities and, finally, two corresponded to severe OSAS with and without comorbidities [4]. In both studies, clinical changes in age, BMI, ODI, AHI and AI were found to be associated with a higher risk of belonging to a more serious phenotype. Despite the strong association between OSAS and obesity, studies have shown that an important number of OSAS patients have a BMI within the normal range [23]. However, this relationship is reinforced by studies concluding that patients with a higher BMI experience frequent ODI periods, resulting in a higher AI and decreased sleep efficiency [24]. Moreover, a 10% increase in weight gain was associated with a huge probability of developing OSAS, and a 10% weight loss predicted a 26% decrease in AHI [25]. Furthermore, as others have noted, both the current study and the study by Vavougios et al. discovered a nonlinear relationship between comorbidity and OSAS severity [26]. This recurrent finding indicates that moderate-to-severe OSAS patients can be furthermore stratified by resiliency against comorbidities [27], albeit studies determining the trajectory of exposure to moderate or severe untreated OSAS are understandably lacking.

There are important differences between the current study and the study by Vavougios et al. that need to be addressed. The current study was a PHC population-based study, as opposed to a study involving OSAS patients recruited from a sleep clinic, where participants reported sleep-related disturbances without a diagnosis of OSAS or other sleeping breathing disorder. Furthermore, in the current study, there were different clinical features and criteria included in the analysis [4].

Few studies have formally characterized the distinct combinations of symptoms and comorbidities in OSAS patients. To our knowledge, the first study that applied cluster analysis and that identified distinct subgroups of OSAS patients was performed by Ye et al. (ISAC study), which only placed emphasis on patients with moderate-to-severe OSAS (AHI ≥ 15 events/h) who were referred to a sleep clinic [28], whereas we also included patients with mild OSAS in a community-based study. The ISAC study was relatively consistent in the severity of the disease and the presence of symptoms, including comorbidities, as they distinguished them in their study population. In contrast to our study, the use of a predetermined definition of comorbidity, such as CCI, is considered a major difference, because CCI excludes hypertension, and cardiovascular disease (CVD) has several different aspects [28]. Recently, Pien GW et al. studied the follow-up data from the ISAC cohort to assess the differences in symptom responses after 2 years of CPAP treatment among the defined clusters. More specifically, they examined the relationship between OSAS clusters and successful CPAP treatment, and they concluded that the clusters with more severe OSAS were more likely to respond to treatment [29]. Keenan et al. (SAGIC study) studied OSAS patients who were middle aged and obese and had predominantly severe OSAS. After using cluster analysis, they found five clusters, where three of them were similar to those of the ISAC study and the remaining two were less symptomatic [30].

The study by Saaresanta et al. studied a huge population in 18 countries who were referred to sleep centers. Four clinical phenotypes were reported based on daytime symptoms; insomnia; and comorbidities, including CVD and anatomical and phycological features. The results are in line with the findings of the ISAC study [31].

A recent clustering study studied a large French population of moderate-to-severe OSAS patients with the same inclusion criteria as in the SAGIC study. They assessed demographics, lifestyle factors, OSAS severity, comorbidities and environmental risk factors. They identified six clusters parallel to those of the SAGIC study, but the absence of insomnia-related symptoms was the key difference. However, the major difference with our study was that OSAS severity measurements (AHI/ODI) were not considered to define subgroups [32].

A smaller study in Italy (198 OSAS patients) identified three clusters in a sample referred to a sleep clinic that were similar to those of our study, and the differences between the clusters were a consequence of OSAS severity, BMI and ESS [33]. The first community-based study was the Wisconsin Sleep Cohort Study, with participants randomly selected from a working population. The study showed a high prevalence of SDB, using PSG in adults [34]. Moreover, a huge study from the Australian PHC system (BEACH) analyzed all adults for OSAS or snoring from 2000 to 2014; however, the data did not reveal if patients were formally tested for OSAS or which path they followed in doing so (direct referral for testing or only after seeing a specialist) [35]. Recently, two Spanish studies, PASHOS and GESAP, studied a program of the PHC system and a sleep unit for OSAS management. Although the protocol that both studies followed was similar to ours, PSG was not used as the standard test for OSAS diagnosis, and the upper age range was much larger in both studies than in our study [36,37].

## 5. Conclusions

The main strength of our study was that we studied a partnership of a PHC system and a sleep clinic to establish OSAS diagnosis and to evaluate the resulting OSAS clusters in the initial population. A potential limitation of our study was that it involved a small sample size, and our population was predominantly male. However, the design of our study was closely associated with clinical practice, and our findings indicate that PHC can be incorporated into the clinical management of OSAS patients in a similar way to that used for other chronic diseases. Our study also corroborated a nonlinear relationship between comorbidity and OSAS severity, indicating that moderate-to-severe OSAS patients can be further stratified by resiliency against comorbidities.

## Figures and Tables

**Table 1:** Sample demographics, questionnaires, and PSG results.

	All Sample	Gender		*p* Value
	*n* = 136	Females	Males	
Age (years)	48 (38, 57.75)	50 (36.8%) 45 (36, 54)	86 (63.2%) 45 (36, 54)	*p* < 0.001
Weight (kgr)	91.50 (84, 105)	88.27 ± 20.32	96.5 (86, 11.3)	*p* = 0.026
Height (cm)	172 ± 9.99	163.5 ± 7.70	176.8 ± 7.33	*p* < 0.001
Body Mass Index (BMI)	32.37 (28.10, 34.08)	32.73 ± 7.59	31.60 (28.03, 33.63)	*p* = 0.204
Low Epworth Sleepiness Scale (ESS)	68 (50%) 8 (6, 9)	29 (58%) 9 (7, 9)	39 (45.3%) 8 (6, 9)	*p* = 0.214
High ESS	68 (50%) 13 (11.25, 15.75)	21(42%) 13 (12, 14.5)	47 (54.7) 13 (11, 16)	*p* = 0.846
Low-Risk Berlin Questionnaire (BQ)	30 (22%)	8 (16%)	22 (25.6%)	
High-Risk BQ	106 (78%)	42 (84%)	64 (74.4%)	
Apnea Hypopnea Index (AHI) Mild	20 (14.7%) 11.70 (10.30, 13.38)	8 (16%) 10.70 (9.20, 13.85)	12 (13.9%) 11.70 (11.40, 13.38)	*p* = 0.333
Moderate AHI	34 (25%) 21.50 (19.85, 25.45)	14 (28%) 20.70 (18.68, 25.55)	20 (23.3%) 22.40 (20.03, 25.53)	*p* = 0.660
Severe AHI	74 (54.4%) 60.40 (41.33, 81.53)	23 (46%) 60.20 (38.30, 70.10)	51 (59.3%) 63.20 (44.70, 86.60)	*p* = 0.382
AHI	31.75 (18.43, 63)	26.55 (14.43, 52.43)	34.95 (20.70, 71.25)	*p* = 0.024
Obstructive Apnea Index (OAI)	7.6 (1.93, 25.33)	4.25 (0.98, 11.38)	10.30 (3.08, 40.43)	*p* = 0.001
Oxygen Desaturation Index (ODI)	24.40 (12.40, 54.53)	19.40 (10.98, 42.43)	27.85 (14.25, 60.98)	*p* = 0.036
Respiratory Arousal Index (RAI)	7.10 (0.5, 28.43)	14.85 (3.55, 30)	4.40 (0.2, 23.85)	*p* = 0.016
Desaturation Arousal Index (DAI)	0.9 (0.3, 2.55)	1 (0.4, 2.33)	0.65 (0.2, 3.1)	*p* = 0.336
Arousal Index (AI)	18.75 (8.33, 36.78)	21.40 (11.15, 34.95)	14.75 (5.98, 45.75)	*p* = 0.228
Charlson Comorbidity Index (CCI)	1 (0, 1)	1 (0, 1)	0 (0,1)	*p* = 0.024

Data are presented as mean ± sd for normally distributed data (height) or median (interquartile ranges) for skewed data (remaining data). Normally distributed data were analyzed by Student *t*-test and skewed data with Mann–Whitney U test.

**Table 2:** Cronbach’s alpha value.

Dimension	Cronbach’s Alpha	Variance Accounted for Total (Eigenvalue)	Dimension	Cronbach’s Alpha
1	0.729	1.946	1	0.729
2	−0.015	0.990	2	−0.015
Total	0.989	2.936	Total	0.989

**Table 3:** Correlation-transformed variables.

	AHI	ODI	CCI (0–37)
AHI	1.000	0.934	0.047
ODI	0.934	1.000	0.100
CCI (0–37)	0.047	0.100	1.000
Dimension	1	2	3
Eigenvalue	1.946	0.990	0.064

**Table 4:** One-way analysis of variance (ANOVA) descriptives.

	Cluster 1	Cluster 2	Cluster 3	*p*-value
	n = 67 (49.3%)	n = 25 (18.4%)	n = 44 (32.4%)	
Males Females	37 (27.2%) 30 (22.05%)	16 (11.76%) 9 (6.61%)	33 (24.26%) 11 (8.08%)	
Age (males) Age (females)	44.11 ± 9.74 49.43 ± 12.54 *p* = 0.017	48.94 ± 12.55 57.00 ± 7.87 *p* = 0.96	42.91 ± 11.15 55.64 ± 8.29 *p* =0.001	
BMI (males) BMI (females)	29.43 ± 4.76 30.71 ± 7.57 *p* = 0.25	33.42 ± 3.75 37.49 ± 6.80 *p* = 0.18	34.14 ± 6.04 35.77 ± 6.03 *p* = 0.46	
ESS (0–24)	10.09 ± 4.13	11.60 ± 3.99	11.23 ± 3.19	*p* = 0.146
AHI	20.08 ± 10.29	44.85 ± 31.08	73.42 ± 20.46	*p* = 0.001
ODI	14.50 ± 8.73	40.47 ± 31.31	61.46 ± 22.01	*p* = 0.001
AI	14.99 ± 10.60	32.69 ± 28.26	42.70 ± 31.58	*p* = 0.001
RAI	7.49 ± 9.01	25.42 ± 29.13	32.36 ± 32.22	*p* = 0.001
DAI	1.02 ± 1.31	1.85 ± 2.47	3.08 ± 3.69	*p* = 0.001
SAI	0.64 ± 1.01	0.81 ± 1.10	0.52 ± 1.04	*p* = 0.541

Data are presented as mean ± standard deviation (SD). ESS = Epworth Sleeping Scale; AHI = apnea index + hypopnea index/h; ODI = Oxygen Desaturation Index; AI = Arousal Index; RAI = Respiratory Arousal Index; DAI = Desaturation Arousal Index; SAI = Snore Arousal Index.

**Table 5:** CCI (0–37) TwoStep Cluster Number crosstabulation.

			TwoStep Cluster Number			Total
			1	2	3	
CCI (0–37)	0	Count	40 _a_	0 _b_	23 _a_	63
		Residual	9.0	−11.6	2.6	
		Std. Residual	1.6	−3.4	0.6	
		Adjusted Residual	3.1	−5.1	1.0	
	1	Count	27 _a_	0 _b_	21 _a_	48
		Residual	3.4	−8.8	5.5	
		Std. Residual	0.7	−3.0	1.4	
		Adjusted Residual	1.2	−4.1	2.1	
	2	Count	0 _a_	14 _b_	0 _a_	14
		Residual	−6.9	11.4	−4.5	
		Std. Residual	−2.6	7.1	−2.1	
		Adjusted Residual	−3.9	8.3	−2.7	
	3	Count	0 _a_	8 _b_	0 _a_	8
		Residual	−3.9	6.5	−2.6	
		Std. Residual	−2.0	5.4	−1.6	
		Adjusted Residual	−2.9	6.1	−2.0	
	4	Count	0 _a_	2 _a_	0 _a_	2
		Residual	−1.0	1.6	−6	
		Std. Residual	−1.0	2.7	−8	
		Adjusted Residual	−1.4	3.0	−1.0	
	8	Count	0 _a_	1 _a_	0 _a_	1
		Residual	−5	8	−3	
		Std. Residual	−7	1.9	−6	
		Adjusted Residual	−1.0	2.1	−7	
Total		Count	67	25	44	136

Each subscript letter denotes a subset of TwoStep Cluster Number categories, whose column proportions do not differ significantly from each other at the 0.05 level.

## Data Availability

Data are available on reasonable request from the corresponding author.
